# Impact of Menopause and Associated Hormonal Changes on Spine Health in Older Females: A Review

**DOI:** 10.3390/cells15020148

**Published:** 2026-01-14

**Authors:** Julia Chagas, Gabrielle Gilmer, Gwendolyn Sowa, Nam Vo

**Affiliations:** 1 Department of Orthopaedic Surgery, University of Pittsburgh, Pittsburgh, PA 15213, USA; jchagas@pitt.edu (J.C.);; 2 Medical Scientist Training Program, University of Pittsburgh School of Medicine, Pittsburgh, PA 15261, USA; gabbygilmer@pitt.edu; 3 Department of Physical Medicine and Rehabilitation, University of Pittsburgh, Pittsburgh, PA 15213, USA

**Keywords:** low back pain, menopause, spine, intervertebral disc degeneration, post-menopausal women, sex hormones

## Abstract

**Highlights:**

**What are the main findings?**

- Menopausal hormone fluctuations are associated with intervertebral disc degeneration, facet joint osteoarthritis, ligamentum flavum hypertrophy, sarcopenia, sympathetic innervation alterations, and systemic inflammation; however, specific mechanisms remain poorly understood.
- Evidence suggests exercise and parathyroid hormone are promising therapeutic options for menopausal low back pain (LBP), while hormone replacement therapy and bisphosphonates seem less promising.

**What are the implications of the main findings?**

- This review highlights critical windows for research to uncover mechanisms and inform improved, targeted treatments for menopause related LBP.

**Abstract:**

Low back pain (LBP) represents a major societal and economic burden, with annual costs in the United States estimated at $90–134.5 billion. LBP disproportionately impacts postmenopausal women relative to age-matched men, suggesting a role for sex-specific biological factors. Although the mechanisms underlying this disparity are not fully understood, hormonal imbalance during menopause may contribute to LBP pathophysiology. This narrative review aimed to elucidate the impact of menopause on LBP, with emphasis on hormonal effects on spinal tissues and systemic processes. A literature search was conducted, followed by screening of titles, abstracts, and full texts of original clinical studies, preclinical research using human or animal samples, and relevant reviews. Rigour and reproducibility were evaluated using the ARRIVE Guidelines and the Modified Downs & Black Checklist. Evidence indicates that menopause is associated with changes in intervertebral discs, facet joint, ligamentum flavum, skeletal muscle, sympathetic innervation, and systemic systems such as the gut microbiome. However, most findings are correlational rather than causal. Evidence supporting hormone replacement therapy for LBP remains inconclusive, whereas exercise and other treatments, including parathyroid hormones, show more consistent benefits. Future studies should focus on causal mechanisms and adhere to rigour guidelines to improve translational potential.

## 1. Introduction

Low back pain (LBP) is defined as pain occurring between the ribs and the buttocks [1] and can be acute, lasting a few days to a few weeks, or chronic, lasting for more than three months [2]. Symptoms can range from a dull ache to sharp, severe pain and may be accompanied by stiffness, reduced mobility, or radiating pain down the legs. LBP is highly prevalent in the US, with approximately 80% of adults experiencing LBP at some point in their lives. Additionally, LBP represents one of the most common reasons for missing work and disability. In 2020, an estimated 619 million people reported LBP globally, and this is estimated to increase to 843 million by 2050 [3]. Additionally, healthcare costs related to LBP are estimated to range between $90 and $134.5 billion in the US alone [4].

A major contributor to LBP is intervertebral disc degeneration (IDD). Clinical assessment of IDD can be performed using magnetic resonance imaging (MRI) and related to patient-reported symptoms through standardized outcome measures. MRI allows for the grading of disc degeneration using the Pfirrmann scale, a widely accepted semi-quantitative system that categorizes discs from Grade I (healthy) to Grade V (severely degenerated) based on parameters such as signal intensity, structural integrity, distinction between the nucleus pulposus and annulus fibrosus, and disc height [5]. These imaging-based indicators of structural deterioration are then compared with clinical evaluations of LBP. Functional impairment due to LBP is often measured with the Oswestry Disability Index (ODI) or Patient-Reported Outcomes Measurement Information System (PROMIS) measures of function, which reflects patients’ self-reported limitations in everyday activities, while pain intensity is assessed using scales such as the Visual Analogue Scale (VAS) or Numeric Rating Scale (NRS) [6]. A consistent association is observed between higher Pfirrmann grades, indicating more severe disc degeneration, and higher ODI scores indicating elevated pain ratings, thereby linking anatomical changes seen on MRI with the patient’s reported pain and functional disability [7]. However, high incidence of disc degeneration is also observed in individuals without LBP, particularly in older populations, complicating assessment of causality [8,9]. In fact, evidence suggests that the differences in genotype expression of pain related genes such as NPY and COMT have an important role in the experience of back pain regardless of the anatomic pain pattern [10,11].

Despite its high prevalence and healthcare burden, LBP remains a significant challenge to treat due, in part, to its multifactorial origin. Specifically, mechanical stress, trauma, genetics, occupational demands, ageing, sex, and gender all contribute to risk for LBP [12,13,14]. When considering sex and gender, evidence consistently suggests that post-menopausal women have a higher prevalence and report more severe LBP compared with age-matched men [15]. This was first observed by Lawrence in 1969. He studied 713 men and 809 women over the age of 35 in the UK and found that 11% of men and 19% of women reported LBP. Among men, the incidence plateaued around the age of 40, but among women, it continued to rise sharply after age 65 [16]. Notably, this study controlled for occupation, housing, and neurological conditions but did not control for BMI or physical activity level. Similarly, the 1996 Occupational Study of Paris surveyed 7010 workers from small factories and found that women experienced a higher incidence and severity of LBP, even when controlling for physical risk factors, high BMI and poor working position [17]. While the mechanisms of this sex-dimorphism are not fully understood, these early findings and others point to a potential role of menopause in modulating the underlying pathology of LBP [18]. Moreover, hormonal changes that occur across the female lifespan, including menarche and pregnancy may also contribute to the pathophysiology of LBP [19]. For instance, studies have shown that the substantial increase in relaxin during pregnancy decreases stiffness in pelvis and spinal ligaments, contributing to loading of other areas of the spine [20].

More recent studies have begun directly interrogating the role of menopause on LBP. In late menopause (i.e., 10+ years beyond the onset of menopause) [21], LBP is the menopause-associated symptom that is the most bothersome, most commonly reported, and most significantly associated with poor quality of life [22,23,24], with 79% of post-menopausal women report LBP [22]. Compared to premenopausal women, women with oophorectomy, in early perimenopause, late perimenopause, or post-menopause reported significantly more LBP in age-adjusted analyses [15,16,17,18,19]. Additionally, one MRI-based study demonstrated that postmenopausal women exhibited significantly more severe lumbar disc degeneration than men and pre- and peri-menopausal women [25]. This association was particularly evident during the first 15 years since menopause; given this time period (i.e., perimenopause) is associated with significant fluctuations in circulating sex hormones, this finding highlights the potential importance of menopause related hormonal shifts in lumbar spine degeneration [25].

In 2021, over one billion women were over the age of 50, accounting for 26% of the population [26]. This number is expected to steadily increase, given the increasing lifespan of our population [26]. Menopause coincides with a critical period of spine degeneration, and it is essential to improve our understanding of the link between menopause, hormonal imbalance, age-associated spine degeneration, and LBP. Understanding this interaction will not only clarify disease mechanisms but also inform future therapeutic strategies aimed at improving spinal health in women before, during, and after the menopause transition. Therefore, the purpose of this paper was to perform a narrative review on the literature aimed at understanding the impact of menopause on the pathophysiology of LBP.

## 2. Materials and Methods

This narrative review synthesized the current evidence on the impact of menopause and female hormones on LBP and degeneration of intervertebral disc and other tissues in the spine. A comprehensive literature search was conducted in PubMed, Scopus, and Web of Science up to July 2025. The following keywords and their combinations were used: menopause, spine, low back pain, intervertebral disc degeneration, and female hormones, including estrogen, progesterone, follicle-stimulating hormone, and luteinizing hormone. These hormones were selected because they play key roles in sexual and reproductive development as well as menopause in women. Boolean operators (AND/OR) were applied to refine results. The reference lists of relevant articles were also screened to identify additional studies.

Publications were restricted to the English language with no time period restriction or restrictions on pre-clinical versus clinical articles. Exclusion criteria included conference abstracts, editorials, case reports, and studies that focused exclusively on osteoporosis or vertebral fractures associated with menopause, as these topics have been thoroughly documented elsewhere [27,28,29].

The selection process was conducted independently by the authors, who screened titles and abstracts, followed by a full-text evaluation. Disagreements were discussed amongst co-authors until a consensus was reached. Data were extracted narratively and organized into the following thematic categories: (1) exploration of the unique factors contributing to how and why LBP manifests differently in women than in men, (2) examination of the underlying mechanisms of LBP in relation to menopause, including both experimental and clinical data on the impact of hormones on spinal tissues and systemic effects, and (3) analysis of hormone replacement therapy (HRT) and other therapies for LBP within the context of menopause, highlighting their efficacies and relevance to LBP.

Given the narrative design, no formal quality assessments or meta-analyses were performed. However, rigour and reproducibility analyses were performed. Specifically, for the clinical original research articles, rigour was scored using a Modified Downs and Black checklist [30], and for preclinical original research articles, rigour was scored using the ARRIVE Guidelines 2.0 (see the Supplementary Materials) [31]. For both assessments, articles received a “2” for that category if it was reported clearly sufficient, “1” if it was unclear if that category was reported sufficiently, and a “0” if it was clearly insufficiently reported.

## 3. Results

### 3.1. Article Inclusion and Rigour and Reproducibility Analysis

**Figure 1:**
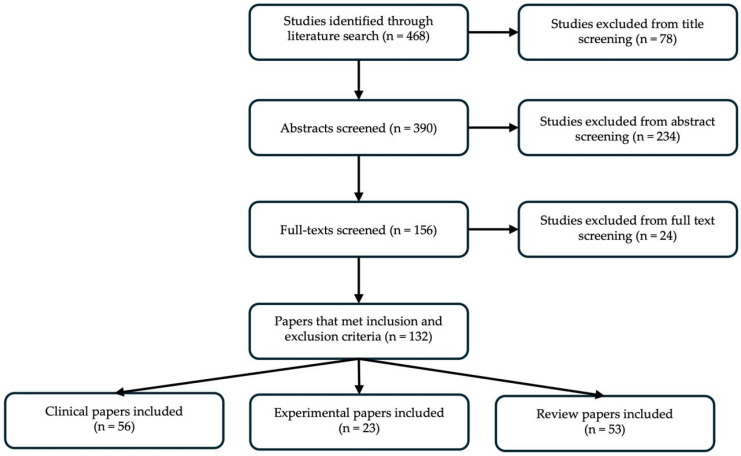
Systematic review workflow. Systematic search of the literature, followed by title, abstract, and full text screening yielded 132 articles to be included in our review.

Our literature search identified 468 articles to be screened for our review. After title, abstract, and full text screening, 132 articles were included in this narrative review. Of these, 56 papers were original clinical research articles, 23 studies were original pre-clinical experimental articles including either human or animal samples, and 53 were review articles (Figure 1).

**Figure 2:**
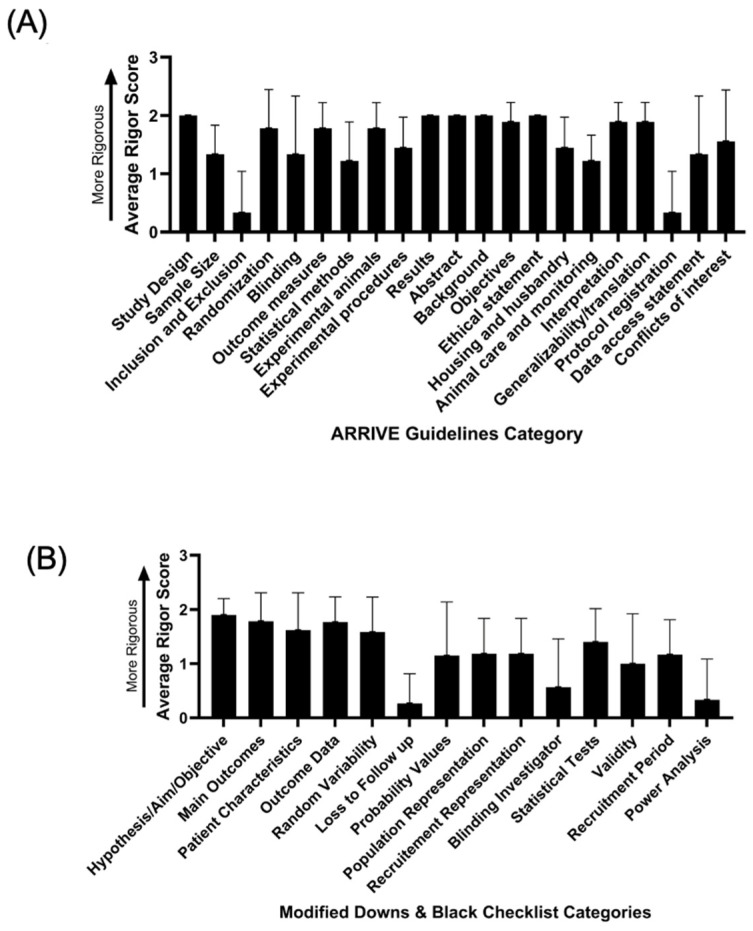
(**A**) ARRIVE guidelines category of the selected preclinical/animal studies. The higher the score, the more rigorous the study was for that category. (**B**) Modified Downs and Black Checklist of the selected clinical studies. The higher the score, the more rigorous the study was for that category.

Among the pre-clinical studies, we found that study design, randomization, outcome measures, experimental animals, results, abstract, background, objective, ethical statements, interpretation, and generalizability/translation were consistently well reported in these articles. However, inclusion/exclusion, sample size calculations, blinding, statistical assumptions, animal care and monitoring, and protocol registration were not well reported in these articles (Figure 2A, Table S1).

Among the clinical studies, we found that study aim, main outcomes, patient characteristics, outcome data, random variability, and the type of statistical tests were consistently and adequately reported in this body of literature. However, information on patients lost to follow-up, probability values, recruitment information, blinding, assumptions for statistical tests, validity of metrics utilized, and power analyses were inadequately reported (Figure 2B, Table S2).

### 3.2. Low Back Pain Pathophysiology

#### 3.2.1. Contributions to LBP from Spinal Tissues

##### Intervertebral Disc

IDD contributes to LBP through biomechanical, biochemical, and structural mechanisms [32]. More specifically, degeneration of the disc nucleus pulposus presents as reduced proteoglycan and water content and results in diminished hydrostatic pressure in the disc. Ultimately, the disc loses height and the ability to distribute loads effectively [33]. The loss of mechanical competence of the disc contributes to abnormal loading of the surrounding facet joints and ligaments and potentially contributes to experienced pain via mechanoreceptor modulation [32]. Additionally, a degenerated disc typically harbours increased senescence and apoptosis of resident cells, elevated inflammatory cytokines and neurotrophic factors, and upregulation of matrix degrading proteases [34]. This pro-inflammatory environment and loss of matrix integrity is thought to contribute to ingrowth of nociceptive nerve fibers and blood vessels into the normally aneural and avascular IDD, further perpetuating pain [35].

Menopause is increasingly recognized as a contributor to IDD. Although men have more risk factors for IDD, women still have more severe disc degeneration; this has been hypothesized to be due to estrogen deficiency inducing vertebral endplate degeneration and impairing nutrient diffusion to the intervertebral discs [36,37]. Indeed, in a cohort study of 1566 women and 1382 men, Lou et al. found that women had higher MRI IDD features than men. This difference remained significant (*p* < 0.05 compared with men and *p* < 0.01 compared with premenopausal and perimenopausal women), even after adjusting for confounding factors such as age, height, and weight. Occupational load, smoking, physical activity, and parity were not included [25,38]. Moreover, in women, the number of years since menopause was highly positively correlated with radiographic signs of lumbar IDD [25,38]. Similarly, in a cohort of age-matched Chinese men and women, Wang et al. found that women had significantly more disc space narrowing, accelerated nucleus pulposus degradation, and worsened vertebral osteoporosis than men [39]. Additionally, estrogen receptors (ERs), such as ERα and ERβ, are expressed within intervertebral discs and appear to mediate discogenic pain. Song et al. [40] demonstrated ER colocalization within Substance P, which is a neurotransmitter and a sensory marker related to pain from the tachykinin neuropeptide family, suggesting that ER signalling may be involved in pain perception among older women. Subsequent work by Song et al. [41] revealed that Substance P mediates estrogen’s modulation of pro-inflammatory cytokine release, reinforcing the link between ER activation, inflammation, and disc degeneration. Moreover, Jin et al. [18] emphasized that ER-mediated pathways play a protective role against IDD by regulating inflammatory and oxidative stress responses. The culmination of this data suggests that the sex-differences observed in IDD may be driven, at least in part, by menopause.

##### Vertebral Endplate

Degeneration of the vertebral bone, more specifically the vertebral endplate, also contributes to LBP. The endplate, which is composed of cartilage and subchondral bone, is a critical interface between the vertebral body and the intervertebral disc, allowing for force transmission during loading and nutrient exchange between disc and vertebral bone [42]. These functions are compromised when the endplate is sclerosed, eroded, or has developed micro-fractures [43]. When the endplate is structurally compromised, inflammatory mediators infiltrate, and nociceptive sensitization occurs in the vertebra itself. Additionally, diminished vertebral bone quality, via decreased bone mass or increased fat infiltration, alters biomechanical loading, which may contribute to pain via mechanoreceptors [44].

In comparing the bony structures between male and female humans, females have, on average, 7.3 degrees greater lumbar lordosis than males [45,46]. This increased curve is thought to be due, at least in part, to women having greater vertebral body wedging in L1-L5 and greater sacral slope [45,46]. When examining the vertebral bodies themselves, the cross-sectional area of the female human skeleton is about 10–25% less than that of male humans, which contributes to greater biomechanical stress under similar loads [47,48,49]. The increased lordosis, diminished cross-sectional area, and increased stress are thought to be contributors to increased LBP via accelerated facet joint degeneration, disc degeneration, and muscle fatigue amongst female humans relative to male humans [47,48]. While there are data in other joints suggesting menopause contributes to osteoarthritis, there is not robust data suggesting this to be the case specifically at the vertebral endplate. Thus, it is unclear if the differences outlined here are due specifically to menopause, skeletal differences in men and women, or alterations in loading throughout the lifetime.

##### Spinal Muscles

Data also suggests that spinal muscle dysregulation, specifically paraspinal and trunk musculature, contributes to LBP. Patients with LBP display increased multifidus muscle atrophy, fatty infiltration, and stiffness which compromises their ability to stabilize the spine [50]. This altered muscle composition creates abnormal muscle tone which may then contribute to abnormal mechanical loading to adjacent structures [51]. Pain-induced reflex inhibition impairs coordinated muscle activation, leading to reduced dynamic stability and increased muscle spasm, thus creating a vicious cycle of more pain [52].

Although there are no studies on the impacts of menopause on spinal muscle specifically, there are data documenting the impact of menopause on skeletal muscle generally. Menopause is associated with accelerated sarcopenia [53], and estrogen receptor signalling has been shown to mediate mitochondrial function, satellite cell activation, and neuromuscular junction integrity [54,55]. The combination of these changes ultimately leads to decreased contractile quality and poor motor control, and in the setting of the paraspinal muscles, may compromise their ability to support the spine. We encourage future research to specifically examine the effects of menopause on spinal muscle, as this previous data suggests a menopausal effect on skeletal muscle is likely.

##### Facet Joint

In a healthy human spine, the facet joints bear approximately 3–25% of the axial load during different activities [56]. However, as the intervertebral disc loses height, this rises to nearly 47% [57]. This increased load contributes to facet joint osteoarthritis via subchondral bone sclerosis, cartilage degeneration, and synovium inflammation [58]. Both the synovium and subchondral bone are richly innervated with both nociceptive and mechanoreceptor fibers, and this mechanical overloading stimulates pain pathways [58].

Menopause is thought to increase risks and incidence of facet joint osteoarthritis. Indeed, several clinical studies have documented increased prevalence of facet joint osteoarthritis with the menopause transition in humans [13]. Mechanistically, Chen et al. found that ovariectomized mice had significant subchondral bone loss, cartilage degeneration, and increased vascular and neural infiltration of facet joints relative to sham mice [59]. In their study, mice treated with 17β-estradiol achieved relative protection against facet joint osteoarthritis, so these effects are thought to be mediated by estrogen signalling though the exact mechanisms are yet to be elucidated. Taken together, this data suggests that menopause is likely a direct contributor to facet joint degeneration.

##### Ligamentum Flavum

Loss of disc height increases mechanical stress on the posterior elements of the spine [60], including the ligamentum flavum. This increased load promotes elastic-fiber fragmentation, increased fibrosis, and ultimately, hypertrophy [61]. As the ligamentum flavum enlarges, it loses elasticity, encroaches on the spinal canal and lateral recesses, leading to less space and mechanical compression of the nerve roots and dural sac [62]. The ligamentum flavum is also another source of inflammation, with studies showing increased release of TGF-β1, cytokines, and neurotrophic factors [63].

There is emerging but limited evidence that menopause may influence hypertrophy of the ligamentum flavum. Histological studies demonstrate that resident fibroblasts from ligamentum flavum tissue contain ERα. In vitro work in resident fibroblasts has shown that 17β-estradiol regulates matrix remodelling by increasing early cell proliferation, increasing MMP-13, and decreasing collagen levels via PI3K signalling [64]. The authors speculate that estrogen deficiency in the context of menopause might then disrupt matrix homeostasis within the ligamentum flavum, thus amplifying the aforementioned hypertrophic mechanisms [65].

#### 3.2.2. Neurologic Contributions to LBP

Alterations in sinuvertebral nerve anatomy and/or sympathetic innervation and firing patterns play a significant role in the development and persistence of LBP [66]. As mentioned previously, nociceptive free nerve endings increase in prevalence in the intervertebral disc, facet joint capsule, and nearby ligaments and are sensitive to the degenerative state of these tissues [67]. Increased inflammation in these tissues also lowers the activation threshold and promotes more spontaneous firing [67]. The dorsal root ganglia are also subject to physical compression with the changing loading patterns and chemical irritation from altered surrounding biochemistry [68].

Evidence suggests that menopause facilitates these changes, particularly with regard to firing. 17β-estradiol has been shown to enhance excitatory synaptic transmission in dorsal horn neurons by potentiating NMDA-receptor mediated signalling, increasing glutamate release, promoting dendritic spine formation, and facilitating long term potentiation for the spinal dorsal horn [69,70]. With menopause, this modulatory control is lost, therefore lowering the threshold for nociceptive activation. In animal models, estrogen deficiency up-regulates NMDAR1 in dorsal root ganglia and is thought to contribute to increased pain sensitivity [71]. Ovariectomized mice also display increased calcitonin gene-related peptide in the spinal dorsal horn, with co-localization with p65 and pro-inflammatory cytokines [72]. These findings suggests that menopausal hormonal changes may foster glial-neural interactions that enhance pain signalling. In addition, one study treated ovariectomized rats with slow-release estrogen 2.5 mg pellet followed by 0.25 mg pellets or placebo suggests that chronic variability in estrogen levels plays a more substantial role in pain modulation than previously appreciated, particularly in the context of heightened nociceptive sensitivity in female rats [73].

#### 3.2.3. Contributions to LBP from Systemic Tissues

Numerous studies have implicated systemic factors, such as inflammation, immune imbalance, and the gut microbiome, as contributors to LBP. For instance, patients with LBP have higher serum interleukin-6 (IL-6) relative to age-matched controls [74]. Increased systemic inflammation weakens the mechanical integrity of the spinal structures by upregulating extracellular matrix degradation, promoting resident cell apoptosis and senescence [75], and encouraging immune cell infiltration. Systemic inflammation also lowers nociceptive activation energy and increases nerve ingrowth via neurotropic stimulation [76]. This global inflammation is thought to be regulated, at least in part, by the gut microbiome, with several recent studies linking dysbiosis with more severe LBP [77,78].

Menopause also increases systemic inflammation. Elevated plasma levels of high-sensitivity C-reactive protein (CRP) and IL-6 were shown to increase the risk of symptomatic lumbar osteoarthritis among postmenopausal women [79], though recent studies in chronic low back pain have suggested this may not be unique to women [80]. More broadly, estrogen deficiency has been mechanistically linked to heightened inflammatory responses contributing to LBP and osteoarthritis pain [81]. In recently post-menopausal women, LBP was correlated with high serum levels of β-endorphin, serotonin, and dopamine [82]. There are also studies linking menopause with altered gut microbiome, thus another possible mechanism by which menopause modulates both inflammation and LBP may be through the gut [83,84].

When examining other medical factors, post-menopausal LBP is highly associated with previous oral contraceptive use [85], early menarche [86,87], pain in the knees and hips [88], headaches [88], high BMI [24,89,90,91], hysterectomy [85,92], previously irregular menstruation [85], severe associated menopausal symptoms [93], having multiple medical comorbidities [89], and having a chronic opioid prescription [89]. Notably though, high BMI, pain in the knees and hips, multiple medical comorbidities, and having chronic opioid prescriptions are also associated with LBP in men, thus, these may be less specific to menopause [94,95,96,97] and more related to ageing and environmental factors. Interestingly, the literature reports that post-menopausal LBP is not associated with the number of children birthed [85], the type of birth undergone [91], or oral contraceptive usage specifically amongst athletes [98].

#### 3.2.4. Sociopsychological Contributions to LBP

Several studies have also examined the relationship between LBP and psychological, social, and behavioural factors. In both men and post-menopausal women, depressed mood [24,89,90,99,100,101], anxiety [90,100,102], lower education level [91,103], impaired physical activity [89,99,104,105], and poor sleep quality [100,106] are associated with more severe LBP. Interestingly, the data on the relationship between smoking and LBP is mixed, with some studies suggesting there is a relationship [89] and other studies suggesting there is no relationship [98]. However, LBP in post-menopausal women is also highly correlated with a history of sexual abuse [93], high perceived stress [90], reduced socializing [24], unemployment [24,107], previous pregnancy [19,85,108], young maternal age at first childbirth [19,85,86], poor interpersonal relationships [107], having disability accommodations [107], consuming alcohol [107], working relatively longer hours [90], and drinking coffee [109]. Notably, these factors are not strongly associated with LBP in men [110,111,112,113,114], thus may be specific to some feature of the female experience. Table 1 summarizes all risk factors outlined above and highlights which ones are likely general LBP risk factors, specific to females but non-hormonal, and specific females and potentially hormone based.

### 3.3. Treatments for Post-Menopausal Low Back Pain

#### 3.3.1. Hormone Replacement Therapy

Evidence supporting hormone replacement therapy for LBP is mixed, and its overall strength is limited because most available studies are retrospective and did not designate low back pain as the primary outcome [13]. Three large (1000+ participants) cross-sectional studies in women aged 55–71 reported higher rates of LBP amongst current HRT users relative to former users or non-users [85,98,115]. In one study, postmenopausal patients with previous hysterectomy received daily oral conjugated equine estrogen (0.625 mg/d) or matching placebo for one-year. Other studies simply asked questionaries about estrogen or progestin-estrogen use without specifying which substance or dose the patients have been using. This relationship is further supported by the HUNT study, which showed the risk of LBP increased with longer duration of HRT [116]. However, a study of approximately 5000 South Korean post-menopausal women suggested that HRT was associated with decreased radiographic signs of degenerative disc disease relative to non-HRT users [117]. Similarly, in a randomized controlled trial including post-menopausal women ages 49–55, women on HRT showed a decrease in the decline of spine mobility relative to placebo, especially when combined with exercise. However, this study showed no effect on LBP or disability symptoms [118]. Similarly, estrogen-progestin treatment was associated with reduced back pain and disability in premenopausal women with low lumbar bone mineral density (BMD) [119]. Moreover, HRT has been suggested to prevent lateral rotatory spondylolisthesis in postmenopausal women [120].

The mechanism by which HRT exerts its effects has begun to be explored in preclinical studies. In ovariectomized rat models, 17β-estradiol attenuated IVD degeneration by inhibiting the NF-κB pathway [121] and by enhancing antioxidant enzyme activity while suppressing autophagy in nucleus pulposus tissues [122]. In addition, HTRA1 derived from osteoclasts in estrogen-deficient rats negatively affected endplate chondrocytes through NF-κB activation, but estradiol treatment counteracted these effects [123]. Hence, preclinical and clinical studies on HRT in mitigating LBP and IDD are limited, and more rigorous research is needed to elucidate the effects of HRT on LBP and IDD.

#### 3.3.2. Exercise

In general, the literature suggests that exercise can help improve post-menopausal LBP. Specifically, two randomized control trials found that post-menopausal women randomized to physical activity reported less LBP than those who were not [124,125]. Several studies have also shown that Pilates decreases LBP and improves quality of life for post-menopausal women [126,127,128]. Castro et al. suggest that these benefits may be due, at least in part, to increased IGF-1/IGFBP-3 ratio and reduction in creatine kinase compared to controls [126]. Although the literature is mixed on whether core stabilization exercises improve LBP, both studies suggest core stabilization improves quality of life in post-menopausal women with LBP [127,129], potentially due to an increase in plasma β-endorphin [130]. Supervised back extensor exercise home programmes have also been shown to decrease LBP, increase back extensor strength, and improve functional mobility [131].

#### 3.3.3. Other Non-HRT Treatments

Beyond HRT, other pharmacological and nonpharmacological interventions have been studied. Although LBP and osteoporosis do not appear to be directly linked, some clinical and preclinical studies have looked at the role of anti-osteoporotic medications to treat LBP. In their review, Bhadouria et al. found that bisphosphonates, intermittent parathyroid hormone, anti-sclerostin antibodies, selective estrogen receptor modulators, and anti-receptor activator of nuclear factor-kappa B ligand inhibitors were associated with improved LBP [132]. Additionally, parathyroid hormone was found to decrease lumbar facet joint degeneration and activate Wnt/β-catenin signalling in ovariectomized rats [51]. Drugs such as Bazedoxifene, a selective estrogen receptor modulator [133], and etidronate combined with alfacalcidol [134] improved lumbar BMD and reduced bone resorption while alleviating LBP in women with osteoporosis. In another set of studies, Kim et al. found improved perceived LBP but worsening BMD with epidural steroid injections amongst post-menopausal women [135].

## 4. Discussion

### Future Perspectives

Significant gaps remain in definitively identifying the mechanisms underlying LBP in women. While the literature implicate menopause as a potential contributor to lumbar IDD and LBP in women, much of the available evidence remains correlational rather than causal [15,136]. Thus, a crucial future direction is to utilize appropriate pre-clinical models that directly investigate the causal relationship between hormonal and reproductive factors and spinal degeneration, instead of depending solely on cross-sectional data [137]. Research into how menopause leads to vertebral endplate degeneration and decreases nutrient diffusion to the intervertebral discs remains underdeveloped and requires further validation. Preclinical studies suggest that estrogen deficiency may impair nutrient supply and accelerate inflammatory and oxidative pathways in the disc [121,122]; however, confirmation of these findings in human populations is very limited. Similarly, while clinical observations link postmenopausal status to accelerated disc degeneration, the temporal sequence and precise pathways need elucidation. Direct serial hormone profiling linked with longitudinal spine MRI in human cohorts remains extremely limited or absent in the published literature. Most human studies use cross-sectional MRI comparisons with menopausal status rather than repeated quantitative hormone measurements tied to imaging changes. Furthermore, most studies have focused almost exclusively on estrogen, with limited attention to the decline of other hormones, such as progesterone, or the fluctuations of gonadotropins like luteinizing hormone (LH) and follicle-stimulating hormone (FSH). Their potential contributions to IDD, adjacent spinal tissues, and pain modulation remain poorly explored, and it is still unclear whether directional changes in estrogen levels—either increases or decreases—are causally linked to the modulation of clinical pain-related syndromes in women [73].

Pharmacological interventions represent another critical area of research. Research is actively exploring whether osteoporosis treatments, including bisphosphonates, parathyroid hormone analogues, and sclerostin inhibitors, could be repurposed to prevent or slow the progression of IDD and associated LBP [138,139]. These approaches are promising but require large-scale trials with spine-specific outcomes to confirm their efficacy; however, current evidence comes primarily from small sample clinical studies and animal models, leaving their translational potential uncertain [140]. Beyond pharmacology, important gaps remain in our understanding of heterogeneity in menopause and treatment response. Natural and surgical menopause may have different effects on spinal health, yet these groups are rarely distinguished in clinical and pre-clinical studies. Lifestyle and genetic factors such as parity, BMI, and physical activity likely influence both risk and treatment outcomes but are understudied. Taken together, these gaps highlight that the field is still in its early stage. Establishing causal mechanisms, validating mechanistic hypotheses, and testing innovative therapies in well-designed, long-term studies will be crucial to move from correlative investigation to actionable prevention and treatment strategies. Ultimately, an integrated framework that includes hormonal, biomechanical, and molecular data may provide the most effective approach to improving spinal health in women during and after the menopausal transition.

Exploring therapeutic strategies may offer new avenues for research, prevention and care. Equally important is the recognition of individual variability, with factors such as age at menopause, type of menopause (surgical versus natural), lifestyle, and genetic predispositions influencing both risk and treatment response. Regarding preclinical evidence, the use of ovariectomy models warrants careful consideration, as these procedures are typically performed in young animals and inherently introduce surgical stress that may confound hormonal and pain-related outcomes. As an alternative, chemically induced ovarian failure has been developed and validated, most notably using 4-vinylcyclohexene diepoxide (VCD), which selectively depletes small ovarian follicles and induces a gradual, menopause-like state [141]. This model more closely recapitulates the endocrine transition observed in natural menopause, can be used in older animals, and has been successfully applied in multiple experimental settings [142].

To overcome the limitations of largely descriptive associations, future research should integrate longitudinal human cohorts with serial estradiol and progesterone profiling and repeated spine MRI alongside complementary animal models with graded hormone replacement, defined as systematically varied hormone doses and exposure patterns, and in vivo imaging. This combined approach would allow hormone trajectories—rather than menopausal status alone—to be directly linked to progressive disc and vertebral degeneration, while controlled preclinical studies would strengthen causal inference and clarify dose- and timing-dependent effects of estrogen and progesterone. By harmonizing imaging and degeneration metrics across species, such a framework has the potential to translate mechanistic insight into targeted, timing-specific interventions that reduce LBP, preserve spinal health and mobility, and ultimately enhance the quality of life for millions of women worldwide.

## Figures and Tables

**Table 1:** Summary of risk factors for general LBP, female non-hormonal risk factors, and female potentially hormone mediated risk factors.

	General LBP Risk Factors	Female Non-Hormonal Risk Factors	Female Potentially Hormone Mediated Risk Factors
Local Structural Changes	IDD [32,33,34,35]	IDD [39]	IDD [18,25,36,37,38,40,41]
	Vertebral endplate degeneration [44]	Vertebral endplate degeneration [45,46,47,48,49]	Facet joint OA [13,59]
	Spinal Muscles [50,51,52]		Ligamentum flavum hypertrophy [64,65]
	Facet joint OA [56,57,58]		Sympathetic Innervation [69,70,71,72]
	Ligamentum flavum hypertrophy [60,61,62,63]		
	Sympathetic innervation [66,67,68]		
	Systemic inflammation [74,75,76,80]		
Systemic Changes	Dysregulated gut microbiome [77,78]		Sarcopenia [53,54,55]
	Pain in the knees and hips [88,95]		Gut microbiome [83,84]
	High BMI [24,89,90,91,94]		
	Comorbidities [89,96]		
Gynecological Conditions			Severe associated menopausal symptoms [93]
			Hysterectomy [85,92]
			Previously irregular menstruation [85]
			Early menarche [86,87]
			Young maternal age at first childbirth [19,85,86]
			Oral contraceptive use [85]
Sociopsychological Conditions	Chronic opioid use [89,97]	High perceived stress [90]	
	Anxiety [90,100,102]	Unemployment [24,107]	
	Lower education level [91,103]	Headaches [88]	
	Impaired physical activity [89,99,104,105]	History of sexual abuse [93]	
	Poor sleep quality [100,106]	Poor interpersonal relationships [107]	
		Having disability accommodations [107]	
		Consuming alcohol [107]	
		Working relatively longer hours [90]	
		Drinking coffee [109]	

## Data Availability

No new data were created or analyzed in this study. Data sharing is not applicable to this article.

## Supplementary Materials

The following supporting information can be downloaded at: https://www.mdpi.com/article/10.3390/cells15020148/s1, Table S1: Individual Modified Downs & Black Checklist for included studies; Table S2: Individual ARRIVE Guidelines score for included studies.

## Abbreviations

The following abbreviations are used in this manuscript:

LBP	Low Back Pain
IDD	Intervertebral Disc Degeneration
US	United States
UK	United Kingdom
MRI	Magnetic Resonance Imaging
ODI	Oswestry Disability Index
VAS	Visual Analogue Scale
NRS	Numeric Rating Scale
HRT	Hormonal Replacement Therapy
BMD	Bone Mineral Density
LSS	Lumbar Spinal Stenosis
ALP	Bone Formation Marker
u-NTx	Bone Resorption Marker
IL6	Interleukin-6
CRP	C-reactive Protein
BMI	Body Mass Index
LH	Luteinizing Hormone
FSH	Follicle-stimulating Hormone
VCD	4-Vinylcyclohexene Diepoxide
